# Development of a Ready‐To‐Use Bioluminescence Immunosensor for the One‐Step Sensitive Detection of Antibodies Against African Swine Fever Virus

**DOI:** 10.1111/1751-7915.70253

**Published:** 2025-10-16

**Authors:** Zhonghui Zhang, Xuesai Li, Qingli Niu, Jinming Wang, Yanghe Liu, Dossêh Jean Apôtre Afayibo, Wenting Chen, Songlin Yang, Hong Yin, Guiquan Guan, Jifei Yang

**Affiliations:** ^1^ State Key Laboratory for Animal Disease Control and Prevention, African Swine Fever Regional Laboratory of China (Lanzhou), Lanzhou Veterinary Research Institute, Chinese Academy of Agricultural Sciences Lanzhou P. R. China; ^2^ Jiangsu Co‐Innovation Center for the Prevention and Control of Important Animal Infectious Disease and Zoonosis Yangzhou University Yangzhou P. R. China

**Keywords:** African swine fever, antibodies, bioluminescence immunosensor, point‐of‐care testing, split‐nanoluciferase system

## Abstract

Rapid and reliable analytical techniques play important roles in various research fields and are particularly crucial for diagnosing infectious diseases in clinical settings. African swine fever (ASF) is a devastating viral pig disease for which no effective vaccine is available. The ongoing ASF pandemic has highlighted the importance of rapid and accurate diagnosis, which enables the timely implementation of control and eradication measures. In this study, a ready‐to‐use bioluminescence immunosensor based on a split‐nanoluciferase (NanoLuc) reporter system was proposed for the one‐step sensitive detection of ASF virus (ASFV) antibodies. Specifically, the NanoLuc subunits SmBiT/LgBiT were each genetically fused to the ASFV p30 protein and protein G and used as probes. The simultaneous binding of the probes to ASFV IgGs induced the reconstitution of functional NanoLuc, which can generate a strong bioluminescent signal output by catalysing the substrate furimazine. This immunosensor allows the rapid and homogeneous detection of ASFV antibodies in solution, requiring only one incubation step of 10 min. This immunosensor also has high sensitivity, high specificity, and a wide dynamic range and is particularly promising for point‐of‐care testing. Comparative analysis of clinical samples validated the reliability and robustness of this approach and demonstrated high consistency with enzyme‐linked immunosorbent assay (ELISA) results (concordance rate: 98.71%). These results suggest that the proposed immunosensor provides an attractive alternative to conventional immunoassays and could be easily repurposed by generating specific probes for antibody detection in other diseases.

## Graphical abstract

A ready‐to‐use bioluminescence immunosensor for rapid, one‐step detection of ASFV antibodies, offering high specificity, high sensitivity, and ease of use, with promising applications for point‐of‐care diagnostics.

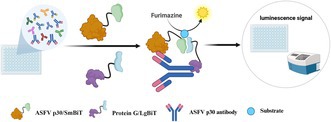

## 1 Introduction

The rapid and accurate measurement of specific analytes in various biological samples is crucial for applications in biological and biomedical science research and clinical practice (Liu et al. 2022). Immunoassays are commonly used bioanalytical tools that determine the presence or concentration of target biomolecules on the basis of specific antigen–antibody interactions and appropriate signal reporting systems (Lim et al. 2023). Conventional immunoassays such as immunofluorescence, enzyme‐linked immunosorbent assays (ELISAs), and chemiluminescence immunoassays (CLIAs) are labour‐intensive, time‐consuming, involve multiple analytical steps, and require specific equipment, making them challenging to use as point‐of‐care systems (Elledge et al. 2021). The lateral flow immunoassay (LFIA) is a rapid, convenient, and easy‐to‐use approach that is applied in point‐of‐care testing, but most LFIA‐based tests lack sufficient specificity and sensitivity (Cui and Zhou 2020).

Recently, bioluminescence‐based immunosensors have attracted increasing attention and represent attractive analytical tools for the determination of various target analytes in bioanalysis applications (Ran and Pu 2023). Among these, the split‐nanoluciferase (NanoLuc) reporter system stands out because of its exceptional sensitivity, specificity, low background signals, and superior signal‐to‐noise (S/N) ratio, making it versatile for studying protein–protein interactions and detecting ternary complex formation both in vitro and in vivo (Liu and Guo 2016; Azad et al. 2021). This platform has been successfully adapted for detecting small molecules, toxins and enzymes (Ding et al. 2022; He et al. 2023; Xie et al. 2024). On the basis of the specific interaction between antigens and antibodies, this technology likewise provides creative opportunities for profiling and discovering pathogens and associated antibodies or other disease biomarkers in clinical diagnostics. To date, split‐NanoLuc assays have been engineered into various formats and applied in the detection of respiratory syncytial virus (RSV), influenza A virus (IAV), *Clostridioides difficile*, and human soluble epoxide hydrolase (sEH) (Adamson et al. 2022; He et al. 2023; Grawe et al. 2024), as well as specific antibodies against SARS‐CoV‐2, Nipah virus and tumour necrosis factor (TNF) (Kim et al. 2021; Yao et al. 2021; Jeremiah et al. 2022; Bergeron et al. 2024).

African swine fever (ASF) is currently the most important and devastating infectious disease affecting swine of all breeds and ages and constitutes the main threat to the global pig industry (Dixon et al. 2020). Although ASF has been documented for more than a century, controlling this disease remains an enormous challenge because of the absence of an effective and safe vaccine (Chen et al. 2024). Rapid and accurate diagnostic tools that can be broadly deployed are crucial for preventing the spread of the disease during the ASF pandemic (Zhu et al. 2024). With the ongoing spread of ASF, serological tests for diagnosing chronic or subclinical infections caused by moderately or low virulent isolates are particularly important because of the silent spread of the disease by viral carriers (Gallardo et al. 2015, 2019). In recent years, ASFV‐antibody‐based tests have been rapidly developed and improved in response to the rapid spread of the disease; however, accurate, sensitive, simple, and convenient tests suitable for early on‐site or point‐of‐care detection remain in high demand to combat the current pandemic and future epidemics, particularly in settings with limited resources (Gallardo et al. 2019). Current detection strategies focus primarily on simplifying testing procedures, reducing reaction time, eliminating sophisticated equipment and technical skills, and providing faster results to support the timely implementation of control measures. In this study, we described a robust ready‐to‐use bioluminescence immunosensor for one‐step detection of ASFV antibodies by exploiting split‐NanoLuc complementation technology. This homogeneous immunosensor exploits the structural and functional characteristics of IgG molecules involving a universal LgBiT‐fusion probe, and a specific SmBiT‐fusion probe binds synchronously to the Fc and Fab regions of IgG, mediating the reconstitution of functional NanoLuc. This assay has a significantly simplified workflow for testing, can be carried out directly in solution, and is simple, rapid, and easy to perform.

## 2 Materials and Methods

### 2.1 Reagents and Sera

The *Ndel* I and *Hind* III restriction enzymes were purchased from New England Biolabs (Ipswich, MA, USA). A Mag‐Beads His‐Tag Protein Purification Kit was acquired from BBI (Shanghai, China). HRP‐conjugated anti‐His tag and HRP‐conjugated anti‐Pig antibodies were obtained from Abcam (Cambridge, MA, USA). The Pierce BCA Protein Assay Kit and Pierce ECL Western Blotting Substrate were purchased from Thermo Fisher Scientific (Waltham, MA, USA). The NanoLuc substrate was acquired from Promega (Madison, WI, USA). The commercial ASFV antibody detection kit was obtained from INGENASA (INGEZIM PPA COMPAC K3; Madrid, Spain). ASFV‐positive and ASFV‐negative serum samples were provided by the African Swine Fever Regional Laboratory of China (Lanzhou). Sera positive for classic swine fever virus (CSFV), porcine reproductive and respiratory syndrome virus (PRRSV), pseudorabies virus (PRV), and porcine circovirus type 2 (PCV2) were obtained from the China Veterinary Culture Collection Center. Positive control serum against foot‐and‐mouth disease virus serotype O (FMDV‐O) was provided by the WOAH/National Foot‐and‐Mouth Diseases Reference Laboratory. Sera positive for *Actinobacillus pleuropneumoniae* (APP) and *Haemophilus parasuis* (HPS) were obtained from the Lanzhou Veterinary Research Institute (LVRI).

### 2.2 Vector Construction

Recombinant vectors containing target fusion gene sequences were constructed via standard molecular biology techniques. To create the fusion probes that bind to the Fc region of IgG, the LgBiT subunit of NanoLuc was genetically fused to the C2 domain of protein G either at the N‐ or C‐terminus via a G/S linker (Gly‐Ser‐Ser‐Gly‐Gly‐Gly‐Gly‐Ser‐Gly‐Gly‐Gly‐Gly‐Ser‐Ser). The antibody‐specific probe that binds to the Fab region of the IgG antibody against ASFV was designed by fusing the SmBiT subunit to the N‐ or C‐terminus of ASFV p30 via a G/S linker. An 8xHis tag was appended in the opposite orientation with respect to the LgBiT and SmBiT subunits for protein purification. The DNA sequences encoding the fusion probes (LgBiT‐G/S linker‐C2‐8xHis tag, 8xHis tag‐C2‐G/S linker‐LgBiT, SmBiT‐G/S linker‐p30‐8xHis tag, and 8xHis tag‐p30‐G/S linker‐SmBiT) were synthesized by GenScript (Nanjing, China) and subsequently cloned into pET30a vectors with *Ndel* I and *Hind* III restriction enzyme sites. All the clones were sequence‐verified by Sangon Biotech (Shanghai, China) before protein expression was performed. The vector design and construction are displayed in the Supporting Information (Figure S1).

### 2.3 Protein Expression and Verification

The pET30a recombinant vectors encoding the LgBiT‐C2 (LN‐C2, where LN represents the N‐terminal fusion with LgBiT), C2‐LgBiT (LC‐C2, where LC represents the C‐terminal fusion with LgBiT), SmBiT‐p30 (SN‐p30, where SN represents the N‐terminal fusion with SmBiT) and p30‐SmBiT (SC‐p30, where SC represents the C‐terminal fusion with SmBiT) fusion proteins were transformed into *Escherichia coli* ( *E. coli* ) DH5α competent cells. Protein expression was carried out as previously described (Zhao et al. 2023). Briefly, the bacteria were cultured in lysogeny broth (LB) medium supplemented with 100 μg/mL carbenicillin under shaking at 200 rpm and 37°C. Until the OD_600_ reached approximately 0.6, expression was induced at 37°C for 6 h in the presence of 0.5 mM isopropyl‐β‐d‐thiogalactopyranoside (IPTG). The culture was subsequently centrifuged at 8000× *g* for 20 min at 4°C, after which the bacterial sediment was collected and lysed via ultrasonication. The target fusion proteins were purified with Mag‐Beads His‐Tag protein purification (BBI, China) according to the manufacturer's protocol. The purity of the recombinant proteins was tested by sodium dodecyl sulfate–polyacrylamide gel electrophoresis (SDS–PAGE), and Western blot was performed with an HRP‐conjugated anti‐His tag antibody (1:3000; Abcam, USA). The protein concentrations were determined with a Pierce BCA protein assay kit (Thermo Fisher Scientific, USA) according to the manufacturer's protocol, and the purified proteins were stored at −80°C until use.

Furthermore, the reactivity of the ASFV antibody‐specific probes was evaluated by Western blot. The SN‐p30 and SC‐p30 fusion probes were resolved on a 12% polyacrylamide gel and then transferred to a PVDF membrane (Millipore, USA). After blocking for 1 h at room temperature in 5% (*w*/*v*) skim milk in phosphate‐buffered saline (PBS) supplemented with 0.05% Tween 20 (PBST), the PVDF membrane was washed five times with PBST (10 min each) and incubated with appropriate dilutions of ASFV‐positive serum. After repeated washes, the membrane was probed with an HRP‐conjugated goat anti‐Pig antibody (1:10000; Abcam, USA). ASFV‐negative serum was used as a control. The protein bands were developed with Pierce ECL Western Blotting Substrate (Thermo Fisher Scientific, USA) and visualised with a Chemi‐Doc imaging system (Bio‐Rad, USA).

### 2.4 Screening of Fusion Sensors

The subunits of NanoLuc can be fused to either the N‐ or C‐terminus of the C2 domain of protein G and ASFV p30, generating four possible sensor combinations: LN‐C2 + SN‐p30, LN‐C2 + SC‐p30, LC‐C2 + SN‐p30, and LC‐C2 + SC‐p30. To study the influence of LgBiT and SmBiT orientation on the ability of the fusion probes to detect ASFV antibodies, ASFV‐positive serum (20 μL) was diluted in PBS and mixed with 100 ng of LgBiT/C2 and 50 ng of SmBiT/p30 fusion sensors in a total volume of 80 μL in a 96‐well white plate. The mixtures were incubated at 37°C for 30 min, after which 20 μL (1/5 total volume) of the furimazine solution (Nano‐Glo Live Cell Assay System, Promega, USA) was added to act as the substrate for the reconstituted NanoLuc. Luminescence signals were recorded with a GloMaxNavigator Microplate Luminometer (Promega, USA). ASFV‐negative serum was included as a control, and each assay was conducted in duplicate. The fusion sensor pair that provided the highest S/N ratio was chosen for further study.

### 2.5 Homogeneous Split‐Luciferase Assay for Antibody Detection

A homogeneous split‐luciferase assay was developed on the basis of the C2‐IgG‐p30 and LgBiT‐SmBiT interactions for the detection of specific antibodies against ASFV. The key elements of this sandwich immunoassay involve the ASFV‐specific antibody (in serum) and the LgBiT/C2 and SmBiT/p30 fusion sensors. To optimise the assay, the dilution factors and concentrations of each component were tested by checkerboard titrations. Briefly, different amounts of LgBiT/C2 (100, 50, 25, 12.5 and 6.25 ng) and SmBiT/p30 (50, 25, 12.5, 6.25, 3.125 and 1.5625 ng) fusion sensors were mixed and incubated separately with ASFV‐positive and ASFV‐negative serum at 37°C for 30 min in a total volume of 80 μL in a 96‐well white plate. The enzymatic reaction was developed by the addition of NanoLuc substrate solution, and the changes in the luminescence signal between positive and negative serum samples were recorded. The optimal amounts of LgBiT/C2 and SmBiT/p30 fusion sensors were determined according to the maximum S/N ratio. The test serum samples have to be diluted to a proper level to minimise the interference caused by the endogenous proteins that may produce a detectable signal in the assay (Kim et al. 2021). The appropriate dilution factor for serum samples was assessed by testing different dilutions of ASFV‐positive serum (0.5, 1, 2.5, 5, 10 and 20 μL in PBS) with the optimal amounts of LgBiT/C2 and SmBiT/p30 fusion sensors. ASFV‐negative serum was included as a control. Similarly, the incubation time (5, 10, 20, 30, 45 and 60 min) for sample testing was optimised on the basis of the maximum S/N ratio mentioned above.

The cutoff value to differentiate the ASFV‐positive and ASFV‐negative serum samples in this homogeneous split‐luciferase assay was determined by testing 191 ASFV‐negative pig serum samples, which were provided by the ASF Regional Laboratory of China (Lanzhou). Each serum sample was tested under the optimal conditions and procedures described above, and the luminescence signal was expressed as the S/N ratio. The cutoff value for the homogeneous split‐luciferase assay was defined as the mean S/N value (*x*) of 191 negative samples plus 3 standard deviations (SDs). Serum samples with S/N values less than ‘*x* + 3 SD’ were considered ASFV‐negative and ASFV‐positive otherwise.

Using the optimal procedures, serum samples positive for CSFV, PRRSV, PRV, PCV2, FMDV‐O, APP, and HPS were assayed by the proposed homogeneous split‐luciferase assay. ASFV‐positive and ASFV‐negative serum samples were included, and the specificity of the assay was assessed on the basis of the determined cutoff value. Furthermore, two‐fold serial dilutions (1:2 to 1:8192) of ASFV‐positive serum were tested by the proposed assay, and the results were compared with those of a well‐characterised commercial ELISA kit (INGEZIM PPA COMPAC K3, INGENASA, Spain) to assess analytical sensitivity.

### 2.6 Sample Analysis and Validation

Clinical pig serum samples were routinely collected from 2020 to 2022 by the ASF Regional Laboratory of China (Lanzhou). To verify the practicality of the homogeneous split‐luciferase assay, 309 clinical pig serum samples were tested simultaneously by the assay and a commercial ELISA kit (INGEZIM PPA COMPAC K3; INGENASA, Spain). This commercial kit is based on a blocking ELISA using a p72‐specific monoclonal antibody and has been well validated for detecting ASFV antibodies (Gallardo et al. 2015). The consistency between the homogeneous split‐luciferase assay and the commercial ELISA kit was determined.

### 2.1 Statistical Analysis

Statistical analyses and curve fitting were conducted using GraphPad Prism version 8.0 (GraphPad Software Inc., La Jolla, CA, USA) or Microsoft Excel (Microsoft Inc., USA). The correlation between the homogeneous split‐luciferase assay and the commercial ELISA kit was analyzed using SPSS software (version 25.0; SPSS Inc., Chicago, IL, USA).

## 3 Results and Discussion

### 3.1 Sensor Design

**FIGURE 1:**
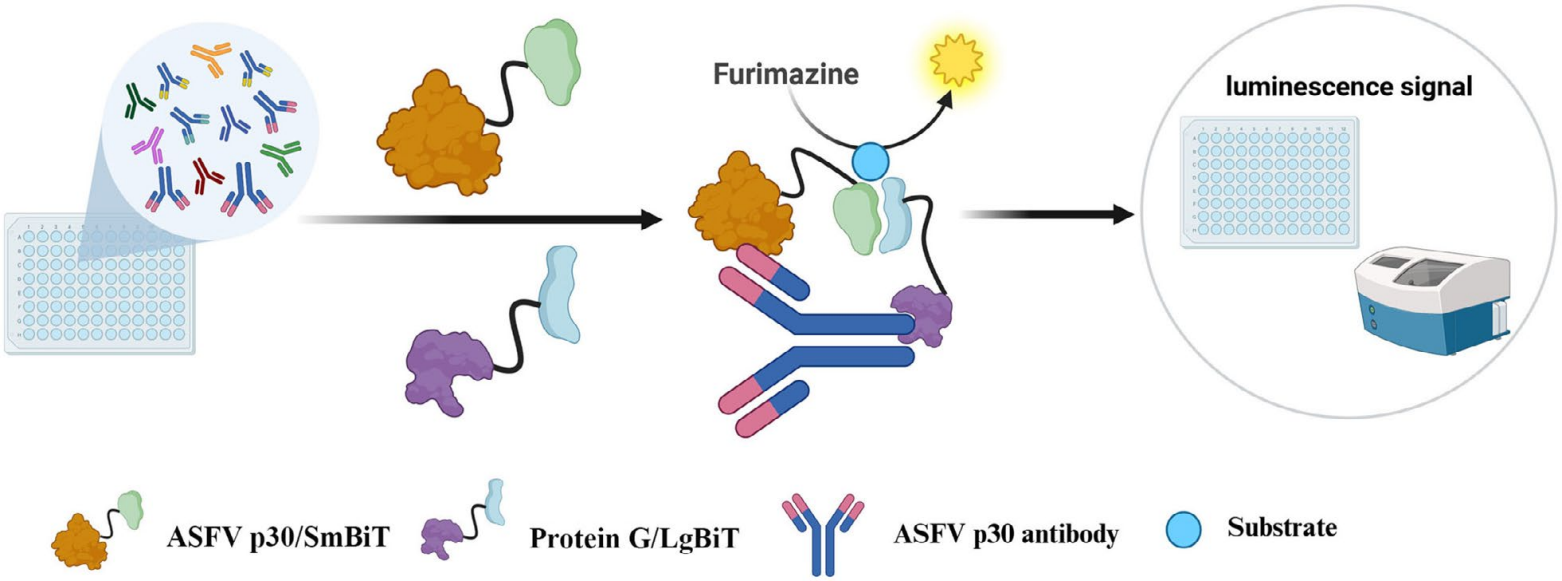
Schematic showing the development of the immunosensor for detecting ASFV antibodies via split‐NanoLuc complementation technology. The ASFV p30/SmBiT and protein G/LgBiT fusion probes of the immunosensor bind to the Fab and Fc regions of IgG antibodies against ASFV p30. They subsequently form a ternary immunocomplex and reconstitute functional luciferase, which can generate a detectable signal by catalysing the substrate furimazine.

In the present study, the split luciferase‐based technique was adapted and employed in the development of a homogeneous immunosensor for the rapid one‐step detection of specific IgGs. The LgBiT and SmBiT subunits of NanoLuc are individually fused to two probes that can accordingly recognise the target analyte (specific IgG) at different binding sites. One probe is designed and constructed by attaching the LgBiT subunit to the C2 domain of protein G (LgBiT/C2), which enables direct conjugation to the Fc region of IgGs (Yao et al. 2021); another probe is generated by fusing the SmBiT subunit to the p30 protein (SmBiT/p30), which can specifically bind to the Fab arms of IgGs against ASFV (Figure 1). Bivalent binding of LgBiT/C2 and SmBiT/p30 to the same IgG molecules results in the formation of a ternary immunocomplex with the probes sandwiching the IgG molecule. Moreover, this binding brings the LgBiT/SmBiT subunits near one another, resulting in the reconstitution of NanoLuc, thereby rapidly generating a strong bioluminescent signal output with furimazine as the substrate (Figure 1). This immunosensor is simple and easy to use and requires only one incubation step (Figure 1). The tested serum samples are diluted and mixed with the two generated probes (LgBiT/C2 and SmBiT/p30) and incubated for some time; afterward, luminescence is immediately measured with a luminometer. This assay can be carried out directly in solution and eliminates the multiple washing and incubation steps required by other serological techniques.

### 3.2 Expression and Verification of the Fusion Sensors

The ASFV p30 protein, one of the most antigenic structural proteins, has been extensively used as a target antigen in ASFV serological diagnostics (Gallardo et al. 2019; Zhou et al. 2023). In this assay, p30 was genetically fused with SmBiT as a specific probe, while the C2 domain of protein G was fused with LgBiT as a universal probe to enable bivalent binding to ASFV IgG antibodies. To minimise steric hindrance, the NanoLuc subunits were connected to p30 or C2 via a glycine/serine (GS)_14_ linker, which is flexible and adequate to allow the complementation of the split NanoLuc reporter fragments (Ni et al. 2019). Engineered fusion probes (SN‐p30, SC‐p30, LN‐C2 and LC‐C2) were expressed in a prokaryotic expression system and further developed as biosensors for detecting IgG antibodies against ASFV. The expression and purity of the fusion sensors were confirmed by SDS–PAGE and Western blot analysis using an HRP‐conjugated anti‐His antibody (Figure S2). Considering that the binding activity of the fusion sensors to the target analyte may be affected by the NanoLuc subunits, the influence of the SmBiT subunit on the binding capacity of p30 derivatives to specific IgGs was subsequently evaluated by Western blot. Both the SN‐p30 and SC‐p30 fusion sensors were specifically recognised by the ASFV antibodies, suggesting that the antibody binding affinity of the fusion sensors was maintained (Figure S3).

### 3.3 Screening of the Fusion Sensors

**FIGURE 2:**
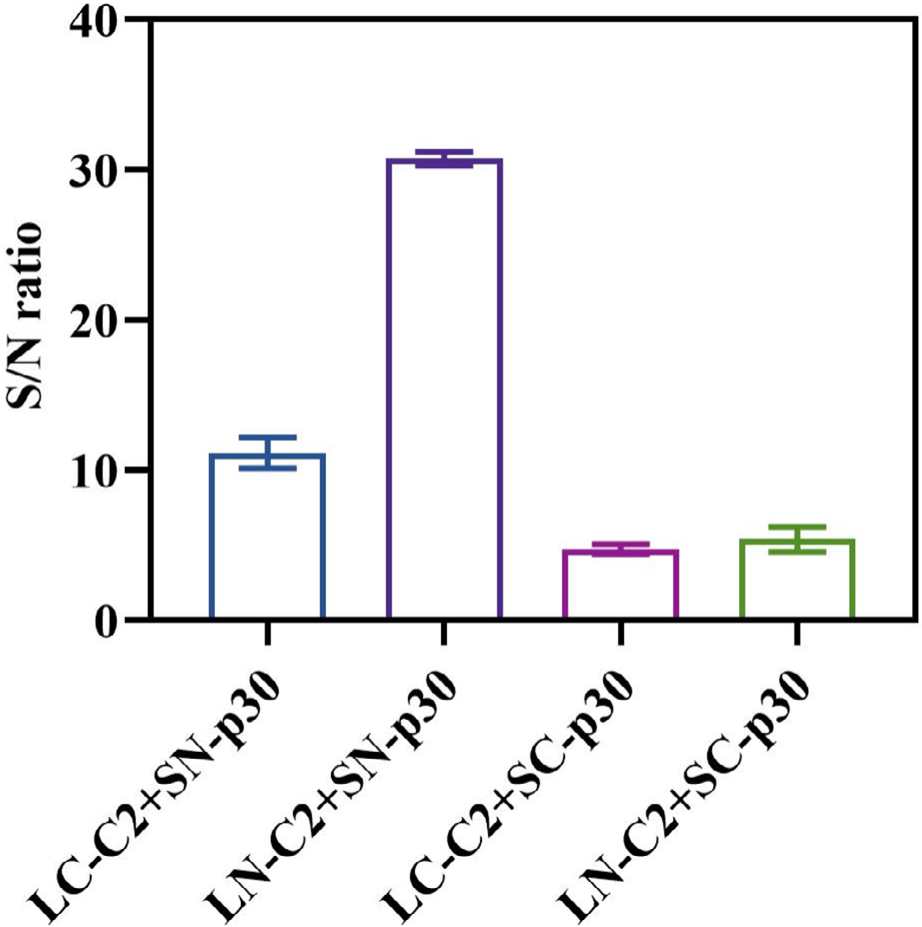
Selection of sensor pairs. Four sensor combinations (LN‐C2 + SN‐p30, LN‐C2 + SC‐p30, LC‐C2 + SN‐p30, and LC‐C2 + SC‐p30) were investigated for their ability to reconstitute functional luciferase by binding with anti‐ASFV antibodies. All sensor pairs produced detectable luminescent signals that differentiated ASFV‐positive sera from negative controls, and the sensor pair LN‐C2 and SN‐p30 achieved the highest signal‐to‐noise (S/N) ratio and was identified as optimal. Data are presented as the means of duplicate tests, and error bars indicate the standard deviations of duplicate tests.

The NanoLuc subunits can fuse to the N‐ or C‐terminus of the analyte binding elements, which may result in variable efficiency in reconstituting the functional enzyme after binding to the target analyte (Elledge et al. 2021). To achieve appropriate split‐luciferase complementation, the influences of LgBiT and SmBiT orientation on the homogeneous immunosensor and their appropriate appending sites were investigated, and different sensor pairs (LN‐C2 + SN‐p30, LN‐C2 + SC‐p30, LC‐C2 + SN‐p30, and LC‐C2 + SC‐p30) were tested for their ability to reconstitute active luciferase in the presence of ASFV antibodies. The results revealed that all the sensor pairs provided detectable signals and differentiated between the ASFV‐positive and ASFV‐negative serum samples, albeit to varying extents (Figure 2). These results suggested that the two fusion sensors, upon target antibody engagement, form a sandwich immunocomplex and bring the LgBiT/SmBiT fragments close to each other, enabling the reconstitution of a functional enzyme. Notably, the greatest amount of signal with the lowest background was observed in the LN‐C2 and SN‐p30 sensor pair, which demonstrated a higher S/N ratio than the other sensor pairs did. Therefore, this pair was chosen for subsequent assays. Furthermore, we observed that the N‐terminal fusion of NanoLuc subunits to both the C2 domain of protein G and the p30 protein was conducive to the reconstitution of an active luciferase in the presence of a specific IgG. These results suggested that the appropriate appending sites of LgBiT and SmBiT to binding elements restore most of their function by mediating conformational changes and producing a high luminescent signal, highlighting the importance of sensor design in the split‐luciferase assay.

### 3.4 Split‐NanoLuc Complementation Assay for the Detection of ASFV Antibodies

**FIGURE 3:**
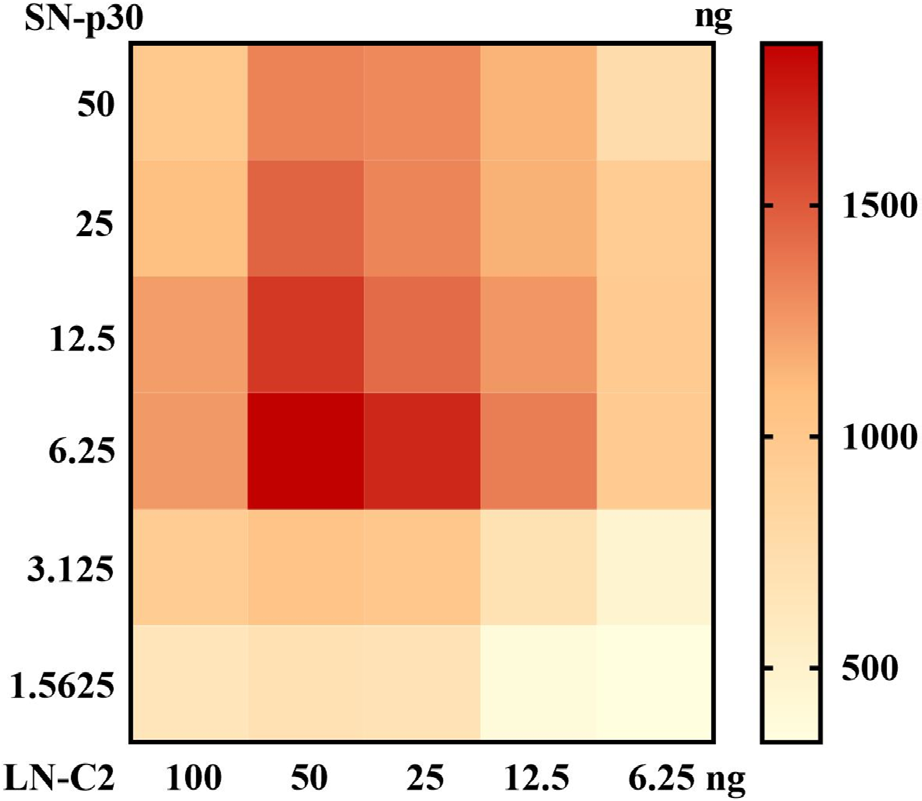
Determination of the optimal doses of the fusion sensors. The maximum luminescence signal and less background were observed at 50 ng of LN‐C2 and 6.25 ng of SN‐p30, yielding the highest signal‐to‐noise (S/N) ratio. The data are presented as the means of duplicate samples.

**FIGURE 4:**
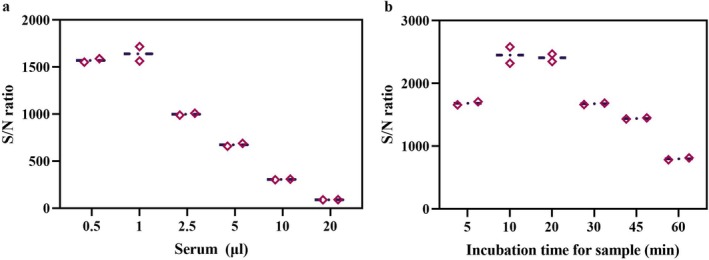
Determination of the optimal reaction conditions. (a) The optimal amount of serum. (b) The optimal incubation time for sample detection. Data is presented as the means of duplicate tests, and error bars indicate the standard deviations of duplicate tests.

As mentioned above, the sensor pair LN‐C2 and SN‐p30 displayed the greatest increase in the IgG‐driven signal, so this sensor pair was applied in a split‐NanoLuc complementation assay for the determination of specific antibodies against ASFV. To obtain the best performance of the proposed assay, the concentrations of the sensor proteins, the dilution factor of the serum, and the reaction time were optimised. We first sought to determine the optimal amounts of the sensor proteins LN‐C2 and SN‐p30 that provided the highest S/N ratio. Serum contains large quantities of IgG antibodies, and the overwhelming majority of these antibodies are not specific for the ASFV p30 protein but can bind to the C2 domain of protein G, which may reduce the sensitivity of the assay. To overcome this issue, different amounts of LN‐C2 (100, 50, 25, 12.5 and 6.25 ng) and SN‐p30 (50, 25, 12.5, 6.25, 3.125 and 1.5625 ng) were tested by a checkerboard titration assay (Figure 3). Excessive amounts of LN‐C2 with respect to SN‐p30 were used in the assay mixture with the purpose of decreasing potential competition by endogenous host IgG. We observed that 50 ng of LN‐C2 and 6.25 ng of SN‐p30 produced the maximum luminescence signal and reduced background noise, resulting in the highest S/N ratio (Figure 3). The luminescence signal of this homogeneous split‐luciferase immunoassay may be affected by the presence of endogenous serum proteins (Kim et al. 2021), and the optimal dilution of serum was further investigated. The results showed that 1 μL (1:100) of serum provided the highest S/N ratio, with a clear positive signal and reduced background noise in this assay (Figure 4a). We also observed that the luminescence signals decreased at high IgG levels, resulting in low S/N ratios (Figure 4a). This ‘hook effect’ (a decrease in signal with high levels of an analyte) has been well documented previously and is caused by the two sensors binding to the analyte separately at high concentrations, rather than binding to the same target molecules to form a sandwich immunocomplex (Ni et al. 2021; Adamson et al. 2022). We also evaluated the effect of incubation time on sensor performance, which is a critical parameter to consider, especially for point‐of‐care testing. The results revealed that the positive signal increased over time, but the background noise increased even more significantly, resulting in low S/N ratios (as demonstrated when the time > 20 min; Figure 4b). A 10 min incubation of the assay mixture (LN‐C2 + SN‐p30 + serum samples) prior to the development of the bioluminescent signal provided the highest S/N ratio, which was identified as the optimal incubation time (Figure 4b).

The determination of a reliable cutoff value is highly important for discriminating between positive (infected) and negative (noninfected) results in diagnostic tests. With the optimal conditions and procedures, 191 samples of pig serum negative for ASFV were tested to establish the cutoff value of the proposed homogeneous split‐NanoLuc complementation assay. The luminescence signal of each sample was recorded and expressed as the S/N value. The mean S/N value (*x*) of the tested negative sera was 1.1057, with a standard deviation (SD) of 0.8685, resulting in a cutoff value of 3.7113 (*x* + 3 SD). Therefore, serum samples with an S/N value ≥ 3.7113 were determined to be positive for ASFV, whereas samples with an S/N value < 3.7113 were regarded as negative.

### 3.5 Cross‐Reaction and Analytical Sensitivity

**FIGURE 5:**
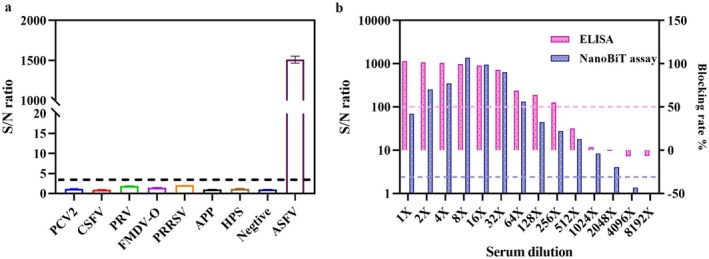
Specificity and analytical performance of the immunosensor. (a) Specificity analysis of the proposed assay by testing sera positive for PCV2, CSFV, PRV, FMDV‐O, PRRSV, APP, and HPS. Apart from ASFV, the signal‐to‐noise (S/N) values of those serum samples were lower than the defined cutoff value of the assay. Data are presented as the means of duplicate tests, and error bars indicate the standard deviations of duplicate tests. (b) Comparison of analytical sensitivity between the proposed assay and ELISA with dilutions of ASFV‐positive samples.

To assess specificity, the proposed split‐NanoLuc complementation assay was used to test several other serum samples from pigs infected with different pathogens, including CSFV, PRRSV, PRV, PCV2, FMDV‐O, APP and HPS. As shown in Figure 5a, apart from that of the ASFV‐positive serum, the S/N values of the other serum samples were significantly lower than the defined cutoff value of the assay, suggesting that they tested negative. These results demonstrated that the developed assay was highly specific for detecting anti‐ASFV IgG antibodies and did not display cross‐reactivity with other pig viruses or bacteria. In this assay, two NanoLuc fragments fused to the sensors LN‐C2 and SN‐p30 bound synchronously to the same IgG molecule against ASFV p30 and formed a ternary complex, ensuring its specificity. Although we have adequately verified the reconstitution of active NanoLuc mediated by the sensors LN‐C2 and SN‐p30 in the presence of specific IgG antibodies, whether the reconstitution of NanoLuc is attainable with different IgG levels is still unclear. We further evaluated the analytical performance of the assay and compared it with that of a commercially available high sensitivity ELISA kit by testing two‐fold serially diluted ASFV‐positive serum, with the dilutions ranging from 1:2 to 1:8192. The results revealed that the maximum dilution of ASFV‐positive serum with an S/N value above the cutoff value was 1:2048, whereas the maximum dilution was 1:256 for the ELISA (Figure 5b). These results suggested that the analytical sensitivity of the split‐NanoLuc complementation assay was approximately eight times greater than that of the ELISA method, demonstrating the superior sensitivity of the assay. The exceptional brightness and stability of reconstituted NanoLuc coupled with the extremely low background signals of its split fragments resulted in outstanding analytical performance of the split‐NanoLuc complementation assay, which enables accurate testing of diluted tested serum samples, decreases the potential interference of endogenous serum proteins, and offers a wide measurement range of IgG levels (Biewenga et al. 2020).

### 3.6 Sample Analysis and Validation

Clinical samples were tested to further evaluate and verify the feasibility of the split‐NanoLuc complementation assay. This assay was applied for the detection of 309 clinical pig serum samples, and the results were compared with those of the commercial INGENASA ELISA kit, which has been well characterized by the European Union Reference Laboratory (EURL) for ASF and has been extensively used for the active surveillance of ASF (Gallardo et al. 2015). The results revealed that both methods identified 133 positive and 172 negative serum samples (305 of the 309 tested samples), with an agreement rate of 98.71% (Table 1). Statistical analysis revealed a high degree of agreement between the split‐NanoLuc complementation assay and the ELISA results (Kappa = 0.9737), suggesting the reliability and robustness of the proposed assay in clinical applications.

**TABLE 1:** Comparison of the proposed assay and a commercial ELISA kit.

Methods and determination indexes	Commercial ELISA kit			Agreement (%)	Kappa	Confidence interval
	Positive	Negative	Total			
The proposed assay						
Positive	133	4	137	98.71	0.9737	0.9611–0.9863
Negative	0	172	172			
Total	133	176	309			

Currently, ELISA remains the most widely used method for antibody detection in ASF serological surveillance because of its high specificity and reliability (Gallardo et al. 2019). However, its complex multistep procedure, time‐consuming nature (typically requiring several hours), and dependence on specialised equipment limit its applicability in point‐of‐care diagnostics (Hosseini et al. 2018). Although LFIAs offer simplicity and field adaptability, they often lack sufficient sensitivity and specificity, particularly in complex matrices such as serum. High background interference in such samples can compromise accuracy and lead to misinterpretation (Budd et al. 2023). In contrast, the split‐NanoLuc complementation assay described in this study addresses these limitations by delivering results within 10 min through a streamlined workflow, making it ideal for rapid on‐site diagnostics. Additionally, this assay significantly reduces background noise, increases detection sensitivity, and enables highly accurate quantification of low‐abundance antibodies. These favourable properties suggest that the split‐NanoLuc complementation assay could be a favourable alternative to traditional techniques for ASFV antibody detection.

## 4 Conclusions

In this study, we developed a homogeneous ‘ready‐to‐use’ bioluminescence immunosensor with a fast readout for the detection of ASFV antibodies using split‐NanoLuc complementation technology. The key factors closely related to immunosensor performance, including sensor design and reaction conditions, were optimized. The N‐terminal fusion of NanoLuc subunits to the universal probe (LN‐C2) and specific probe (SN‐p30) is beneficial for IgG‐driven NanoLuc complementation with a strong positive signal and S/N ratio. Compared with traditional immunoassays, this immunosensor demonstrates high sensitivity and specificity, a wide dynamic range, and good performance. Notably, the proposed approach can be performed directly in solution in a simple, wash‐free, and mix‐and‐measure format and can be adapted easily for the detection of antibodies against other human and animal pathogens by simply replacing the specific sensor accordingly.

## Author Contributions

**Zhonghui Zhang:** methodology, investigation, formal analysis, writing – original draft. **Xuesai Li:** investigation, formal analysis, writing – review and editing. **Qingli Niu:** methodology, investigation, writing – review and editing. **Jinming Wang:** methodology, investigation, formal analysis. **Yanghe Liu:** investigation, formal analysis. **Dossêh Jean Apôtre Afayibo:** formal analysis, writing – review and editing. **Wenting Chen:** investigation, formal analysis. **Songlin Yang:** investigation, formal analysis. **Hong Yin:** methodology, writing – review and editing. **Guiquan Guan:** methodology, writing – review and editing. **Jifei Yang:** conceptualization, methodology, investigation, formal analysis, writing – original draft, writing – review and editing.

## Ethics Statement

All the animals were handled following the Animal Ethics Procedures and Guidelines of the LVRI, CAAS (approval number: LVRIAEC‐2023‐043).

## Conflicts of Interest

The authors declare no conflicts of interest.

## Supporting information


**Figures S1‐S3:** mbt270253‐sup‐0001‐FigureS1‐S3.docx.

## Data Availability

Data are available from the corresponding author upon request.
