# Supplementary material for: Development of a Ready‐To‐Use Bioluminescence Immunosensor for the One‐Step Sensitive Detection of Antibodies Against African Swine Fever Virus
Source: Microb Biotechnol. 2025 Oct 16;18(10):e70253. doi: 10.1111/1751-7915.70253 (PMC12529227; doi:10.1111/1751-7915.70253)
Supplement: Supplementary file 1 — Figures S1‐S3: mbt270253‐sup‐0001‐FigureS1‐S3.docx. [file MBT2-18-e70253-s001.docx]

**Supplementary Information**

**Development of a ready-to-use bioluminescence immunosensor** **the one-step sensitive detection of antibodies against African swine fever virus.**

Zhonghui Zhang^1#^, Xuesai Li^1#^, Qingli Niu^1^, Jinming Wang^1^, Yanghe Liu^1^, Dossêh Jean Apôtre Afayibo^1^, Wenting Chen^1^, Songlin Yang^1^, Hong Yin^1, 2^, Guiquan Guan^1^ and Jifei Yang^1*^

1, State Key Laboratory for Animal Disease Control and Prevention, African Swine Fever Regional Laboratory of China (Lanzhou), Lanzhou Veterinary Research Institute, Chinese Academy of Agricultural Sciences, Xujiaping 1, Lanzhou, Gansu, 730046, P. R. China

2, Jiangsu Co-Innovation Center for the Prevention and Control of Important Animal Infectious Disease and Zoonosis, Yangzhou University, Yangzhou 225009, P. R. China

*** Corresponding authors:** Assoc. Prof. Jifei Yang, Email: [yangjifei@caas.cn](mailto:yangjifei@caas.cn), Tel: +86-931-8342671, Fax: +86-931-8340977

#Zhonghui Zhang and Xuesai Li contributed equally to this article.


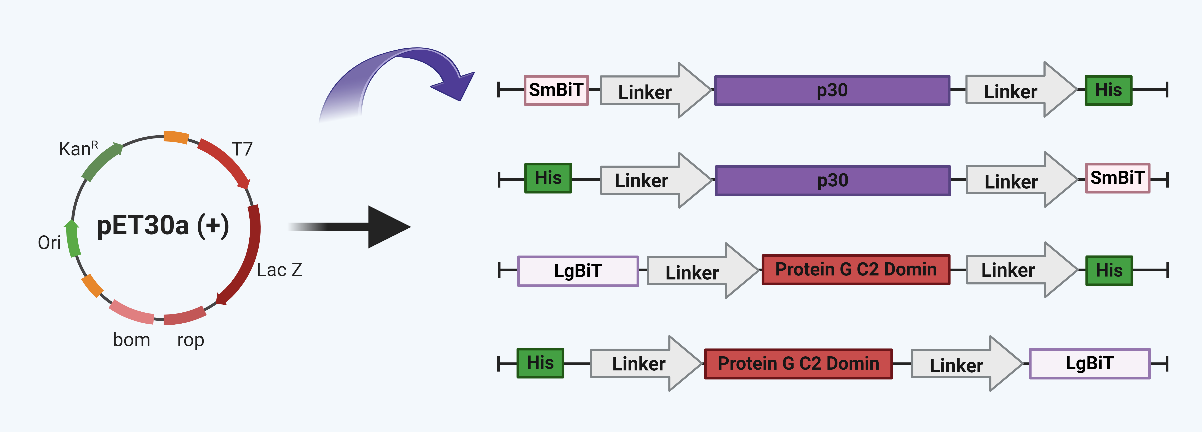


**Figure S1.** Structural schematic diagrams of NanoLuc subunit fusion probes, including SmBiT-p30 (SN-p30, SN represents N-terminal fusion with SmBiT) and p30-SmBiT (SC-p30, SC represents C-terminal fusion with SmBiT), LgBiT-C2 (LN-C2, LN represents N-terminal fusion with LgBiT), C2-LgBiT (LC-C2, LC represents C-terminal fusion with LgBiT). The linker between NanoLuc subunits and p30/C2 represent the sequence of GSSGGGGSGGGGSS, and the linker between His tag and p30/C2 represent the sequence of GGGS.


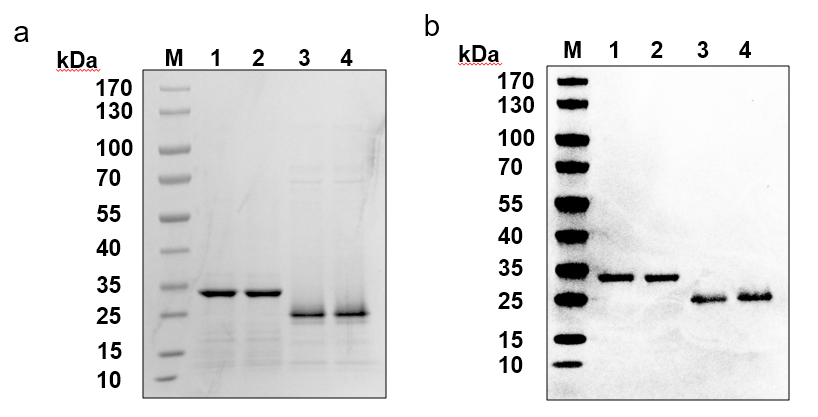


**Figure S2.** SDS-PAGE and Western blot analysis of the expression of LgBiT and SmBiT fusion proteins. SDS‒PAGE analysis of four LgBiT and SmBiT fusion probes (a). Western blot analysis of four LgBiT and SmBiT fusion probes (b). M: protein molecular marker; lane 1-4: SmBiT-p30 (SN-p30) and p30-SmBiT (SC-p30), LgBiT-C2 (LN-C2), and C2-LgBiT (LC-C2).


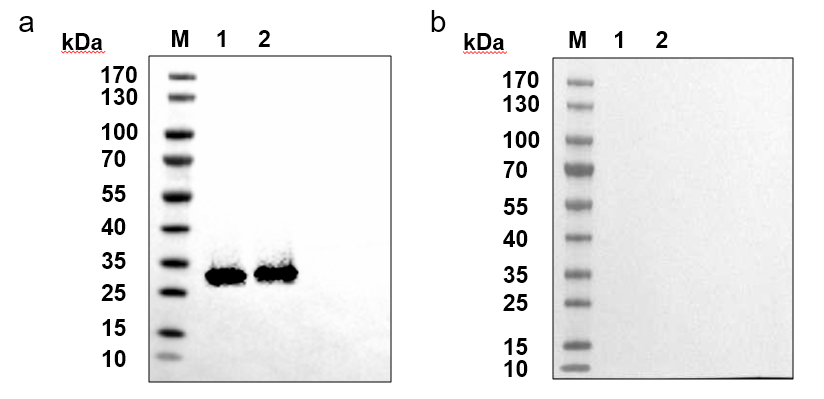


**Figure S3.** Western blot analysis of the immunoreactivity of SmBiT-p30 (SN-p30) and p30-SmBiT (SC-p30). The SN-p30 and SC-p30 fusion sensors were probed by the positive serum and a HRP-conjugated Goat Anti-Pig antibody (a). The SN-p30 and SC-p30 fusion sensors were probed by the negative serum and an HRP-conjugated Goat Anti-Pig antibody (b). M: protein molecular marker; lane 1: SmBiT-p30 (SN-p30), lane 2 p30-SmBiT (SC-p30).
